# Structural Characterization of New Peptide Variants Produced by Cyanobacteria from the Brazilian Atlantic Coastal Forest Using Liquid Chromatography Coupled to Quadrupole Time-of-Flight Tandem Mass Spectrometry

**DOI:** 10.3390/md13063892

**Published:** 2015-06-18

**Authors:** Miriam Sanz, Ana Paula Dini Andreote, Marli Fatima Fiore, Felipe Augusto Dörr, Ernani Pinto

**Affiliations:** 1 Faculty of Pharmaceutical Science, University of São Paulo, Avenida Lineu Prestes 580, Bl-17-05508-900 São Paulo, SP, Brazil; E-Mails: miriam.sanz.roldan@usp.br (M.S.); fadorr@usp.br (F.A.D.); 2 Center for Nuclear Energy in Agriculture, University of São Paulo, Avenida Centenário 303, 13400-970 Piracicaba, SP, Brazil; E-Mails: apdini@cena.usp.br (A.P.D.A.); fiore@cena.usp.br (M.F.F.)

**Keywords:** cyanobacteria, peptides, mass spectrometry, *Brasilonema*, *Nostoc*, *Desmonostoc*, structural elucidation, HPLC-ESI-QTOF-MS, aeruginosin, cyanopeptolin, anabaenopeptolin

## Abstract

Cyanobacteria from underexplored and extreme habitats are attracting increasing attention in the search for new bioactive substances. However, cyanobacterial communities from tropical and subtropical regions are still largely unknown, especially with respect to metabolite production. Among the structurally diverse secondary metabolites produced by these organisms, peptides are by far the most frequently described structures. In this work, liquid chromatography/electrospray ionization coupled to high resolution quadrupole time-of-flight tandem mass spectrometry with positive ion detection was applied to study the peptide profile of a group of cyanobacteria isolated from the Southeastern Brazilian coastal forest. A total of 38 peptides belonging to three different families (anabaenopeptins, aeruginosins, and cyanopeptolins) were detected in the extracts. Of the 38 peptides, 37 were detected here for the first time. New structural features were proposed based on mass accuracy data and isotopic patterns derived from full scan and MS/MS spectra. Interestingly, of the 40 surveyed strains only nine were confirmed to be peptide producers; all of these strains belonged to the order Nostocales (three *Nostoc* sp., two *Desmonostoc* sp. and four *Brasilonema* sp.).

## 1. Introduction

Cyanobacteria represent a rich source of bioactive compounds, many of which remain unexplored. To date, antibacterial, antiviral, antifungal, anticancer, immunosuppressive, protease inhibitory, and other pharmacological properties have been described for the secondary metabolites isolated from these microorganisms [1,2,3,4,5,6,7]. Structurally, these compounds are primarily of a peptide nature [1,3,8] and are mainly synthesized via non-ribosomal peptide synthetases (NRPS) or a combination of NRPS and the polyketide synthetase (NRPS/PKS) [3]. Most of these cyanopeptides can be grouped into seven families based on conserved substructures [3]: anabaenopeptins, cyanopeptolins, cyclamides, microcystins, microginins, aeruginosins, and microviridins. Particular interest arises from cyanobacteria growing in extreme environments, since these underexplored habitats may harbor a microbial diversity able to synthesize a unique variety of secondary metabolites [9].

The Atlantic Forest is a continental biome that extends primarily along the Atlantic coast of Brazil and also includes small portions of Argentina and Paraguay [10,11]. Considered one of the richest regions in the world in terms of biodiversity and endemism, this forest is also one of the most threatened regions [12]. With respect to the microorganisms that inhabit this region, little is known [13]. Particularly, referring to cyanobacteria, the available information is typically focused on diversity and descriptions of species [14,15,16,17] and scarce information about secondary metabolite production is available [18]. Cyanobacteria inhabiting the phyllosphere (leaf surface) are exposed to hostile conditions such as low availability of nutrients, large temperature range, osmotic stress, and high incidence of ultraviolet rays [19], representing a little explored community of extremophilic organisms with potential for production of novel bioactive compounds.

Due to the development in instrumentation and technology, mass spectrometry (MS) has rapidly become a fundamental tool for characterizing large biomolecules such as proteins and peptides [20,21]. The development of electrospray ionization (ESI) and matrix-assisted laser desorption/ionization (MALDI) in conjunction with the introduction of multi-stage and hybrid analyzers was decisive for the success of MS analysis of biomolecules [20]. Time-of-flight (TOF) is one type of mass analyzer that finds wide application in this field and can provide high-resolution spectra for protein and peptides. Particularly when combined with other types of mass analyzer, such as the quadrupole mass analyzer forming the hybrid quadrupole time-of-flight (QTOF), valuable peptide or amino acid sequence information can also be obtained. Liquid chromatography (LC) is a typical inlet method for MS analysis and provides an extra dimension of separation. LC can be coupled with ESI (online/offline) or MALDI (offline). Matrix-assisted laser desorption ionization-time-of-flight-mass spectrometry (MALDI-TOF-MS) has been employed in the identification of many known and new cyanopeptides directly from cyanobacteria [22,23,24,25,26]. Applications of liquid chromatography (LC) coupled to quadrupole time-of-flight mass spectrometry (QTOF-MS) have also been reported [27,28,29].

In this study, the peptide profiles of 40 strains of cyanobacteria isolated from the phyllosphere of four native species of plants in the coastal forest of Southeastern Brazil [30] (Table 1) were investigated using LC-ESI-Q-TOF-MS with positive ion detection and revealed a high production potential in the heterocytous strains. Accurate masses and isotope patterns for both precursor and product ions were used to propose the planar structures of the observed peptides.

**Table 1:** Cyanobacteria included in the present study.

Strain	Order	Family	Genus ^a^	Source ^b^
CENA353	Chroococcales	Xenococcaceae	*Chroococcidiopsis* sp.	*Mn*-SV
CENA367			*Chroococcidiopsis* sp.	*Ee*-SV
CENA351		Hydrococcacceae	*Pleurocapsa* sp.	*Mn*-SV
CENA350	Pseudanabaenales	Pseudanabaenaceae	*Leptolyngbya* sp.	*Mn*-SV
CENA355			*Leptolyngbya* sp.	*Mn*-SV
CENA359			-	*Gg*-Pi
CENA364			*Leptolyngbya* sp.	*Gg*-Pi
CENA370			*Oculatella* sp.	*Ee*-SV
CENA374			*Leptolyngbya* sp.	*Ee*-Pi
CENA372			*Leptolyngbya* sp.	*Ee*-Pi
CENA375			*Leptolyngbya* sp.	*Ee-Pi*
CENA377			*Leptolyngbya* sp.	*Ee-Pi*
CENA378			*Leptolyngbya* sp.	*Ee-Pi*
CENA384			-	*Go-Pi*
CENA385			-	*Go-Pi*
CENA387			*Leptolyngbya* sp.	*Go*-Pi
CENA354	Nostocales	Microchaetaceae	-	*Mn*-SV
CENA352		Nostocaceae	*Nostoc* sp.	*Mn*-SV
CENA356			*Nostoc* sp.	*Mn*-SV
CENA357			*Nostoc* sp.	*Gg-Pi*
CENA358			*Nostoc* sp.	*Gg-Pi*
CENA362			*Desmonostoc* sp.	*Gg-Pi*
CENA363			*Desmonostoc* sp.	*Gg-Pi*
CENA365			*Desmonostoc* sp.	*Gg-Pi*
CENA368			*Nostoc* sp.	*Ee-SV*
CENA369			*Nostoc* sp.	*Ee-Pi*
CENA371			*Desmonostoc* sp.	*Ee-SV*
CENA373			*Nostoc* sp.	*Ee-SV*
CENA376			*Nostoc* sp.	*Ee-Pi*
CENA379			*Nostoc* sp.	*Ee-SV*
CENA380			*Desmonostoc* sp.	*Ee-SV*
CENA383			*Desmonostoc* sp.	*Go-Pi*
CENA386			*Desmonostoc* sp.	*Go-Pi*
CENA388			*Nostoc* sp.	*Go-Pi*
CENA389			*Nostoc* sp.	*Go-Pi*
CENA360	Nostocales	Scytonemataceae	*Brasilonema* sp.	*Gg-Pi*
CENA361			*Brasilonema* sp.	*Gg-Pi*
CENA366			*Brasilonema* sp.	*Gg-Pi*
CENA381			*Brasilonema* sp.	*Ee-Pi*
CENA382			*Brasilonema* sp.	*Ee-Pi*

^a^ Identity criteria for the determination of genus was established in ≥95% when comparing with 16S rRNA genes between the surveyed cyanobacteria and the sequences deposited in the *GenBank* (NBCI). ^b^ Plant species: *Ee*: *Euterpe edualis*; *Mn*: *Merostachys neesii*; *Gg*: *Garcinia gardneriana*; *Go*: *Guapira opposita*. Localization Pi: Picinguaba; SV: Santa Virginia sites, in Parque Estadual da Serra do Mar, São Paulo, Brazil.

**Table 2:** LC-QTOF data of the peptides from the hydromethanolic extracts of cyanobacteria isolated from the Brazilian Atlantic Forest.

No	Compound ^a^	RT (min)	[M + H]^+^ *m/z*	Molecular Formula ^b^	Error (ppm)	mSigma	Detected in Strains CENA									Reference
							*Nostoc* sp			*Brasilonema* sp				*Desmonostoc* sp		
*aeruginosins*																
1	Hpla-Leu-(Hex)-OHChoi-Agma	17.2	753.4042	C_35_H_55_N_6_O_12_	−1.7	13.2	352			360		381	382			this study
2	Hpla-Leu-(Hex)Choi-Agma	18.0	767.3828	C_35_H_55_N_6_O_13_	−1.6	2.1	352	358		360		381	382			this study
3	Hpla-Leu-(Gluc)-OHChoi-Agma	18.2	737.4044	C_35_H_55_N_6_O_11_	−1.2	2.2	352	358		360		381	382			this study
4	Hpla-Leu-(Hex,But)-OHChoi-Agma	20.0	823.4470	C_39_H_63_N_6_O_13_	−2.7	1.0	352	358		360		381				this study
5	Hpla-Leu-(Gluc,But)-OHChoi-Agma	20.6	837.4253	C_39_H_61_N_6_O_14_	1.0	13.0	352	358		360						this study
6	Hpla-Leu-(Hex,Hexan)-OHChoi-Agma	22.5	851.4780	C_41_H_67_N_6_O_13_	2.3	2.0	352	358	369	360	361	381	382			this study
7	Hpla-Leu-(Gluc,Hexan)-OHChoi-Agma	23.1	865.4560	C_41_H_65_N_6_O_14_	−0.8	2.8	352	358	369	360	361	381	382			[37]
8	Hpla-*N*MeLeu-(Gluc,Hexan)-OHChoi-Agma	23.8	879.4717	C_42_H_67_N_6_O_14_	−0.9	18.9	352			360						this study
9	Hpla-Leu-(Gluc,Hep)-OHChoi-Agma	25.6	879.5078	C_43_H_71_N_6_O_13_	−0.5	10.7		358		360		381				this study
10	Pla-dehydroLeu-(Gluc,Hexan)-OHChoi-Agma	25.1	849.4596	C_41_H_65_N_6_O_13_	0.9	9.1	352	358		360		381				this study
11	Hpla-Leu-(Gluc,Oct)-OHChoi-Agma	26.1	893.4872	C_43_H_69_N_6_O_14_	−0.6	0.8	352	358		360		381	382			this study
*anabaenopeptins*																
12	Lys-CO[Lys-Ile-Hph-*N*MeAsn-Phe]	24.0	850.4818	C_43_H_64_N_9_O_9_	0.4	7.2									386	this study
13	Arg-CO[Lys-Ile-Hph-*N*MeAsn-Phe]	24.4	878.4864	C_43_H_64_N_11_O_9_	2.2	11.0									386	this study
14	Lys-CO[Lys-Ile-MeHph-*N*MeAsn-Phe]	24.8	864.4972	C_44_H_66_N_9_O_9_	0.7	1.8									386	this study
15	Arg-CO[Lys-Ile-MeHph-*N*MeAsn-Phe]	25.2	892.5023	C_44_H_66_N_11_O_9_	1.8	4.0									386	this study
16	Lys-CO[Lys-Ile-EtHph-*N*MeAsn-Phe]	26.0	878.5122	C_45_H_68_N_9_O_9_	1.4	2.0									386	this study
17	Arg-CO[Lys-Ile-EtHph-*N*MeAsn-Phe]	26.3	906.5196	C_45_H_68_N_11_O_9_	−0.4	5.0									386	this study
18	Phe-CO[Lys-Val-Hty-MeAla-Hty]	31.1	858.4405	C_45_H_60_N_7_O_10_	−1.1	2.5	352									this study
19	Phe-CO[Lys-Ile-Hty-MeAla-Hty]	31.8	872.4544	C_46_H_62_N_7_O_10_	1.0	5.9	352									this study
20	Val-CO[Lys-Ile-Trp-MeAla-Phe]	34.1	803.4425	C_42_H_59_N_8_O_8_	3.2	5.2				360			382			this study
21	Val-CO[Lys-Ile-Trp-MeAla-Phe]	34.6	803.4417	C_42_H_59_N_8_O_8_	4.1	9.6				360			382			this study
22	Leu-CO[Lys-Ile-MeHph-*N*MeAsn-Phe]	34.8	849.4872	C_44_H_65_N_8_O_9_	−0.4	3.5									386	this study
23	Phe-CO[Lys-Val-Hph-MeAla-Hty]	35.0	842.4416	C_45_H_60_N_7_O_9_	−3.1	1.9	352									this study
24	Phe-CO[Lys-Ile-Hph-MeAla-Hty]	35.8	856.4561	C_46_H_62_N_7_O_9_	5.0	1.6	352									this study
25	Phe-CO[Lys-Ile-MeHph-*N*MeAsn-Phe]	35.4	883.4695	C_47_H_63_N_8_O_9_	2.0	12.6		358								this study
26	Leu-CO[Lys-Ile-EtHph-*N*MeAsn-Phe]	36.1	863.5028	C_45_H_67_N_8_O_9_	0.3	4.0									386	this study
27	Phe-CO[Lys-Ile-EtHph-*N*MeAsn-Phe]	36.7	897.4858	C_48_H_65_N_8_O_9_	1.2	3.3		358								this study
*cyanopeptolins*																
28	Mdhp-Gln[Thr-Leu-Ahp-Leu-*N*Me-Cl-Tyr-Leu]	27.7	1002.5031	C_48_H_73_ClN_9_O_12_	3.0	12.0								371	386	this study
29	AcPro-Gln[Thr-Leu-Ahp-Val-*N*Me-OMe-Tyr-Val]	28.9	984.5381	C_48_H_74_N_9_O_13_	2.0	13.5									386	this study
30	AcPro-Gln[Thr-Leu-Ahp-Val-*N*Me-Cl-Tyr-Val]	29.1	1004.4831	C_47_H_71_ClN_9_O_13_	2.3	5.9								371	386	this study
31	AcPro-Gln[Thr-Leu-Ahp-Leu-*N*Me-Tyr-Leu]	29.9	998.5531	C_49_H_76_N_9_O_13_	2.7	9.9									386	this study
32	AcPro-Gln[Thr-Leu-Ahp-Val-*N*Me-Cl-Tyr-Leu]	30.5	1018.5011	C_48_H_73_ClN_9_O_13_	0.0	4.8								371	386	this study
33	AcPro-Gln[Thr-Leu-Ahp-Val-*N*Me-OMe-Tyr-Val]	31.0	984.5398	C_48_H_74_N_9_O_13_	0.3	18.4								371	386	this study
34	AcPro-Gln[Thr-Leu-Ahp-Leu-*N*Me-Cl-Tyr-Leu]	31.6	1032.5170	C_49_H_75_ClN_9_O_13_	0.3	4.9								371	386	this study
35	AcPro-Gln[Thr-Leu-Ahp-Val-*N*Me-OMe-Tyr-Leu]	32.1	998.5549	C_49_H_76_N_9_O_13_	0.8	15.8								371	386	this study
36	AcPro-Gln[Thr-Leu-Ahp-Leu-*N*Me-OMe-Tyr-Val]	32.7	998.5564	C_49_H_76_N_9_O_13_	−0.7	2.6								371	386	this study
37	AcPro-Gln[Thr-Leu-Ahp-Leu-*N*Me-OMe-Tyr-Leu]	33.9	1012.5737	C_50_H_78_N_9_O_13_	2.4	8.0								371	386	this study
38	PrPro-Gln[Thr-Leu-Ahp-Leu-*N*Me-OMe-Tyr-Leu]	35.2	1026.5858	C_51_H80N_9_O_13_	1.1	26.7								371	386	this study

^a^ Hpla: hydroxy-phenyl lactic acid; Pla: phenyl lactic acid; OH: hydroxy; Hex: hexose; Gluc: glucuronic acid; But: butanoic acid; Hexan: hexanoic acid; Hep: heptanoic acid; Oct: octanoic acid; Me: methyl; Et: ethyl; [] cyclo; Ac: acetyl; Pr: propanoyl; Mdhp: methyl-dehydroproline; *N*Me-Cl-Tyr: *N-*methyl-3-chloro-tyrosine; *N*Me-OMe-Tyr: *N-*methyl-*O*-methyl-tyrosine.

## 2. Result and Discussion

**Figure 1:**
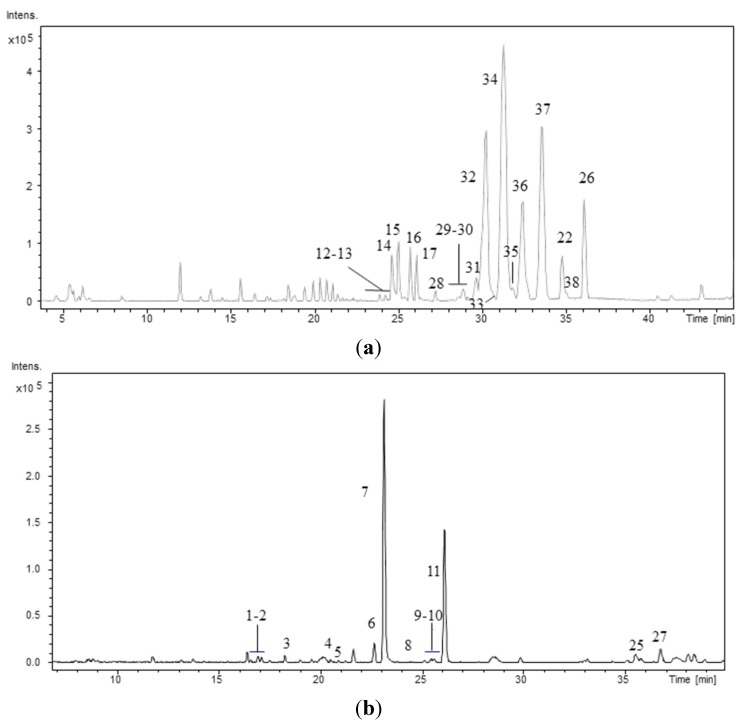
Base Peak Chromatogram of the hydromethanolic extract of the strains (**a**) *Desmonostoc* sp. CENA386 and (**b**) *Brasilonema* sp. CENA358. Conditions as described in experimental section.

The peptide profiles of the 40 strains isolated from the Atlantic Forest (CENA350-389) were investigated using LC/DAD/ESI/QTOF/MS/MS. As an example, Figure 1 shows the chromatographic profiles of two strains: one *Brasilonema* sp. and one *Desmonostoc* sp. The retention times (RT), protonated molecules ([M + H]^+^), molecular formula provided for the experimental *m/z*, and error and millisigma (mSigma) values for the major peaks are summarized in Table 2. Mass accuracy was below 5 ppm for all the detected compounds. Peak identification was performed based on the data presented in Table 2, Table 3, Table 4, Table 5, Table 6, Table 7, Table 8, Table 9, Table 10, Table 11, Table 12, Table 13 and Table 14 and based on previously published data [23,24,26,31,32,33,34,35,36,37,38,39,40]. This approach allowed the elucidation of the planar structures of 38 peptides, including 10 new aeruginosins, 16 new anabaenopeptins, and 11 new cyanopeptolins. Among the surveyed strains the heterocytous strains *Nostoc* sp. CENA352, CENA358, and CENA369, *Brasilonema* sp. CENA360, CENA361, CENA381, and CENA382 and *Desmonostoc* sp. CENA386 and CENA371 were identified as producers of cyanopeptides. Cyanopeptides were not detected in the extracts of the remaining 31 cyanobacterial strains under our experimental conditions.

### 2.1. Aeruginosins

Seven strains belonging to the orders Nostocales (*Nostoc* sp. CENA352, CENA358, and CENA369; and *Brasilonema* sp. CENA360, CENA361, CENA381, and CENA382) were found to produce aeruginosins (**1–11**). Aeruginosins are linear tetrapeptides that contain the unusual amino acid 2-carboxy-6-hydroxyoctahydroindol (Choi) in the central position and typically contain an arginine derivative at the *C*-terminus [3]. The *N*-terminal position is occupied by a 3-(4-hydroxyphenyl) lactic or phenyl lactic acid, which can be acetylated, brominated, chlorinated, or sulfonated [41,42,43,44]. A small hydrophobic amino acid (leucine or isoleucine) is typically present in position 2. Additionally, hydroxylation, sulfation, and chlorination of the Choi moiety have also been observed [45]. These peptides are potent inhibitors of serine proteases, and this bioactivity is largely related to *C*-terminal modifications [46]. This family of compounds has been reported to be produced by cyanobacteria of the genera *Microcystis* [41,42,43,44,47], *Planktothrix* [24], *Nodularia* [48], and *Nostoc* [37].

**Figure 2:**
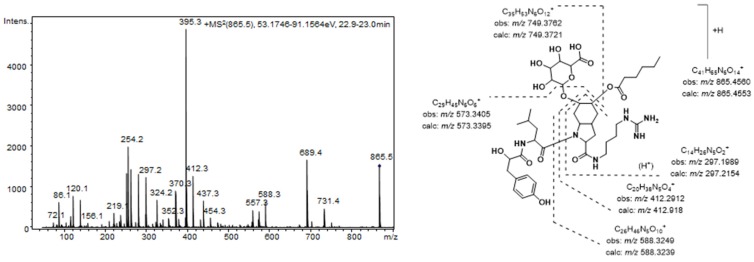
Product ion spectrum for [M + H]^+^ of compound **7** and its predicted fragmentation pattern. Conditions as mentioned in the experimental section.

The aeruginosins found in these extracts were characterized by closely related structures, most of which were common to both *Nostoc* and *Brasilonema* producer species. The most prominent peak detected in the MS chromatogram of these extracts (**7**) was assigned to the aeruginosin 865 (*m/z* 865.4565 [M + H]^+^) [37]. This compound, which was recently isolated from a terrestrial cyanobacterium belonging to *Nostoc* sp., was structurally characterized as containing both a fatty acid and a carbohydrate attached to the Choi moiety [37]. Figure 2 shows the product ion spectra of this aeruginosin. A collision energy of 70 eV was necessary to obtain a spectrum with abundant and intense product ions. Consistent with the existence of an agmatine (Agma) residue in the molecule, the *C*-terminal ions (*m/z* 588.3249, 412.2912, and 297.1989) and the corresponding satellite ions, which were produced via ammonia or water loss (*m/z* 571.2985, 395.2657, 279.1820, and 261.1717) dominated the spectrum. Additionally, the ions generated by the cleavage of the glycosidic acid and/or the ester bond established the sugar and the lipid acids as glucuronic acid and hexanoic acid, respectively. The presence of ions at *m/z* 18 u higher (*m/z* 156.1035 and 138.0866) than the diagnostic ions that are typically generated from the Choi residue (*m/z* 140 and 122) indicated the dihydroxylation of the indole ring in this amino acid and were key fragments for the detection of other aeruginosin congeners.

A total of 10 additional aeruginosins that shared conserved substructures (**1**–**6**, **8**–**11**) and yielded [M + H]^+^ at *m/z* 753.4042 (**1**), 767.3828 (**2**), 737.4044 (**3**), 823.4470 (**4**), 837.4253 (**5**), 851.4780 (**6**), 879.5078 (**8**), 879.5078 (**9**), 849.4596 (**10**), and 893.4872 (**11**) were identified as new congeners. The product ion spectra of these aeruginosins were similar to that of compound **7**, allowing us to elucidate their structures based on a comparison of spectra (Table 3, Table 4 and Table 5).

**Table 3:** Product ion spectra data for compounds **4**–**7**, **9**, and **11**.

Product Ion Assignment ^a^	4 (*m/z*)	5 (*m/z*)	6 (*m/z*)	7 (*m/z*)	9 (*m/z*)	11 (*m/z*)
Leu immonium	86.0967	86.0947	86.0907	86.0945	86.0906	86.0932
OH-Choi immonium ion − H_2_O	138.0791	138.0866	138.0910	138.0912	138.0938	138.0903
OH-Choi immonium ion	156.1035	156.1035	156.1033	156.1026	-	156.1031
OH-Choi-Agma − NH_3_ − H_2_O + H	261.1693	261.1707	261.1799	261.1717	261.1791	261.1709
OH-Choi-Agma − H_2_O + H	279.1802	279.1801	279.1799	279.1820	279.1796	279.1828
OH-Choi-Agma + H	297.1925	297.1885	297.1882	297.1989	297.1880	297.1878
(*R*^1^)-*O*-Choi-Agma + H − NH_3_	367.2348	367.2342	395.2672	395.2657	409.2803	423.2969
(*R*^1^)-O-Choi-Agma + H	384.2618	384.2617	412.2803	412.2912	426.3113	440.3226
(*R*^1^, *R*^2^)-OH-Choi-Agma+ H − NH_3_	- ^b^	543.2599	-	571.2985	-	599.3216
(*R*^1^, *R*^2^)-OH-Choi-Agma + H	546.3176	560.2818	574.3454	588.3249	-	616.3618
Hpla-Leu-OHChoi-Agma + H − H_2_O	573.3423	573.3422	573.3417	573.3405	-	573.3409
Hpla-Leu-(*R*^1^)OH-Choi-Agma	661.3949	661.3909	689.4266	689.4250	703.4423	717.4543
Hpla-Leu-(*R*^2^)OH-Choi-Agma	735.3952	749.3722	735.3945	749.3762	-	-
Hpla-Leu-(*R*^1^, *R*^2^)-OH-Choi-Agma	823.4470	837.4253	851.4780	865.4560	879.5078	893.4872

^a^ *R*^1^ aliphatic lipid acid linked to hydroxyChoi in position 5: butanoic acid for compounds **4** and **5**; hexanoic acid for **6** and **7**; heptanoic acid for **9** and octanoic acid for **11**. *R*^2^ sugar linked to hydroxyChoi in position 6: hexose for compounds **4** and **6**; and glucuronoic acid for **5** and **7**; ^b^ - not detected.

**Table 4:** Product ion spectra data for compounds **1**–**3**.

Product Ion Assignment ^a^	1 (*m/z*)	3 (*m/z*)	2 * (*m/z*)
Leu immonium	86.0908	86.0908	86.0967
OH-Choi immonium ion − H_2_O	138.0867	138.0941	122.1066 *
OH-Choi immonium ion	156.0957	156.1036	140.1034 *
OH-Choi-Agma − NH_3_ − H_2_O	261.1592	- ^b^	-
OH-Choi-Agma − NH_3_	297.1920	297.1928	263.1868 *
OH-Choi-Agma	314.2143	314.2255	281.1914 *
*R*^2^-OH-Choi-Agma − NH_3_	-	473.2107	443.2511 *
*R*^2^-OH-Choi-Agma	476.2717	490.2454	460.2740 *
Hpla-Leu-OHChoi-Agma	591.3360	591.3204	575.3841 *
Hpla-Leu-(*R*^2^)-OH-Choi-Agma	753.4042	767.3828	737.4044 *

^a^ *R*^2^ sugar linked to hydroxyChoi in position 6: hexose for compounds **1** and **2**; and glucuronoic acid for **3**; ^b^ - not detected; * structure lacking the 5-hydroxylation in Choi moiety.

**Table 5:** Product ion spectra data for compounds **8** and **10**.

Product Ion Assignment ^a^	8 (*m/z*)	10 (*m/z*)
*X* immonium	100.1148	86.0906
OH-Choi immonium ion − H_2_O	138.1248	138.0863
OH-Choi immonium ion	- ^b^	156.0952
OH-Choi-Agma − NH_3_ − H_2_O	261.1689	261.1687
OH-Choi-Agma − H_2_O	279.1797	279.1796
OH-Choi-Agma − NH_3_	297.1771	-
OH-Choi-Agma + H	-	-
(*R*^1^)-OH-Choi-Agma − NH_3_	395.2544	395.2659
(*R*^1^)-OH-Choi-Agma + H	412.2930	412.2928
(*R*^1^, *R*^2^)-OH-Choi-Agma − NH_3_	571.3340	571.2881
(*R*^1^, *R*^2^)-OH-Choi-Agma + H	588.3205	588.3238
*Z*-*X*-(*R*^1^)OH-Choi-Agma + H	-	673.4238
*Z*-*X*-(*R*^1^)-OH-Choi-Agma + H	703.4398	-
*Z*-*X*-(*R*^1^, *R*^2^)-OH-Choi-Agma + H	879.4717	849.4596

^a^ *R*^1^: butanoic acid linked to hydroxyChoi in position 5; *R*^2^: glucuronoic acid linked to hydroxyChoi in position 6. *X* aminoacid in second position: methyl-leucine for compound **8** and leucine for **10**; *Z* phenyl alkanoic acid in position 1: hydroxylphenyl lactic acid for compound **8** and phenyllactic acid for **10**; ^b^ - not detected.

In this sense, a structure similar to that of aeruginosin 865 was proposed for compounds **5**, **9**, and **11** except for the fatty acid esterifying position 5 of the Choi moiety. In this position, butanoic, heptanoic, and octanoic acid were proposed for each compound, respectively. These changes were clearly evidenced by the sequence of ions containing the aforementioned fatty acids (*m/z* 384.2617, 543.2599, 560.2818, and 661.3909 for compound **5**; *m/z* 409.2803 and 703.4423 for compound **9**; and *m/z* 423.2969, 599.3216, 616.3618, and 717.4543 for compound **11**). On the other hand, structural differences between the pairs of compounds **4**, **5** and **6**, **7** were attributed to Choi-glycosylation.A similar product ion spectra, which differed only in the ions generated by the cleavage of the glycosidic bond (*m/z* 546.3176, 574.3454, and 735.3952/735.3945) suggested that the glucuronic acid in compounds **5** and **7** was replaced by a hexose in compounds **4** and **6**.

Compounds **1** and **3**, which also showed structures similar to compounds **4** and **6** and **5**, **7**, **9**, and **11**, respectively, were distinguished by the lack of fatty acids in their structures (Table 4). These peptides could be biosynthetic intermediates of the respective fatty acid-containing aeruginosins. Along with these compounds, an oxygen-deficient variant of compound **1** (**2**) was also detected; this structural difference was likely due to the absence of the Choi 5-hydroxylation.

Finally, two other structural variants of compound **7** were also detected (**8** and **10**). For compound **8**, methylation of the amino acid in the second position was suggested based on the mostly conserved product ion spectrum and the presence of a fragment ion at *m*/*z* 100.1148 (*N*MeLeu immonium). Similarly, for compound **10**, a phenyl lactic acid was proposed for the *N*-terminus instead of a hydroxyl-phenyl lactic acid (Table 5).

From a biomedical point of view, the pharmacological potential of these new aeruginosin variants must be evaluated. As mentioned above, aeruginosins typically exhibit antithrombotic activity, making these compounds interesting candidates for the development of anticoagulant drugs [46]. Additionally, all of these compounds are structurally similar to aeruginosin-865, which has exhibited remarkable anti-inflammatory activity [37]. Evaluations of the bioactivity of these compounds could provide insights into the structure-activity relationship of this class of aeruginosins.

### 2.2. Anabaenopeptins

Fifteen compounds produced by *Nostoc* sp. CENA352, *Brasilonema* sp. CENA360, and *Desmonostoc* sp. CENA386 were characterized as new anabaenopeptin analogs (**12**–**27**). Protonated molecules in the mass range of anabaenopeptins and an important loss of the amino acid in the side-chain position were the diagnostic criteria used to classify these peptides [23]. These new anabaenopeptins were characterized based on empirical formulae, product ion spectra, and previously described sequences [23,24,26,34,36].

Anabaenopeptins are hexapeptides that contain a ring of five amino acids. Position 2 is always occupied by d-Lys, which both closes the ring with the amino acid at position 6 and establishes a ureido link with the amino acid in position 1, giving rise to a side chain. Positions 4 and 5 are typically occupied by aromatic and methylated amino acids, respectively. Position 3 has been reported to be occupied primarily by valine or isoleucine/leucine and less frequently by methionine [3]. Various biological activities have been described for these structures, including inhibition of protein phosphatase [49], carboxypeptidases A [50,51] and U [52], and other protease inhibitory activity [53]. To date, 30 anabaenopeptins have been isolated from many different cyanobacteria genera [8,54] (*Anabaena* [55,56], *Aphanizomenon* [51], *Lyngbya* [57], *Microcystis* [58], *Oscillatoria* [53,59], *Planktothrix* [60], and *Schizothrix* [61]) and also from marine sponges [62,63].

**Figure 3:**
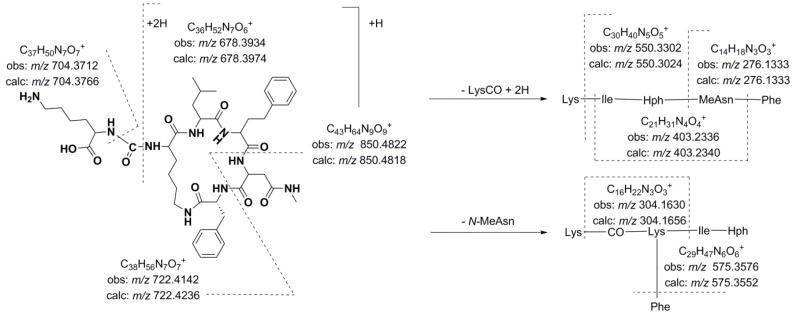
Predicted fragmentation pattern for compound **12**.

**Figure 4:**
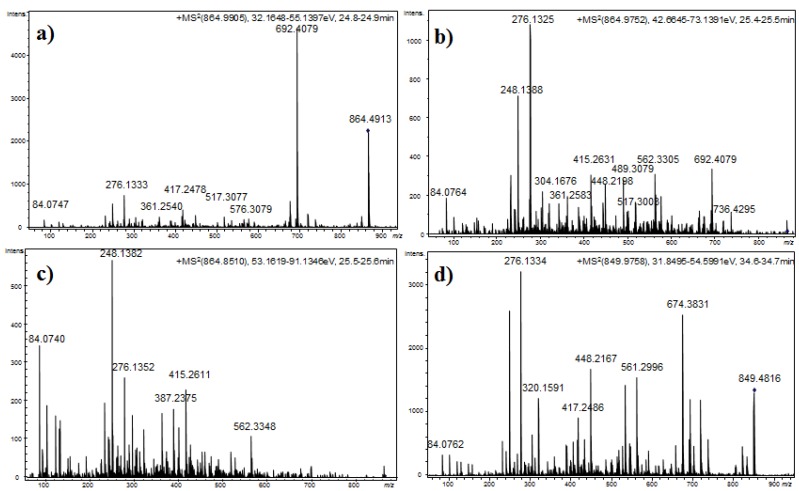
Product ion spectra for [M + H]^+^ of compound **14** at (**a**) CE: 35 eV; (**b**) CE: 50 eV; and (**c**) CE: 70 eV; and (**d**) compound **21** at CE 35 eV.

The detected anabaenopeptins could be grouped according to their structural features. Among these compounds, 10 anabaenopeptins that shared similar structural characteristics were detected in *Desmonostoc* sp. CENA360 and *Brasilonema* sp. CENA386 (**12**–**17**, **22**, **25**–**27**). The representative fragmentation pathways and spectra of these compounds are described in Figure 3 and Figure 4, and the assignments of the principal ions are shown in Table 6, Table 7, Table 8 and Table 9. Since the nature of the exocyclic amino acid varies among them, two different fragmentation patterns were observed for these compounds depending on the nature of the amino acid side chain. Extensive fragmentation was observed due to the absence of polar and basic residues in this chain. These anabaenopeptins commonly incorporate the amino acid *N*-methyl asparagine (*N*-MeAsn) in position 5, and on several occasions, either methylation or ethylation was postulated for the homovariant amino acid in position 4.

**Table 6:** Product ion spectra data for compounds **12**, **14**, and **16**.

Product Ion Assignment ^a^	12 (*m/z*)	14 (*m/z*)	16 (*m/z*)
Lys fragment	84.0768	84.0748	84.0760
MeAsn immonium ion	101.0702	101.0670	101.0649
Phe immonium ion	120.0738	120.0780	120.0793
*Z* immonium ion	- ^b^	148.1125	162.1259
MeAsn-Phe − CO + H	248.1406	248.1400	248.1355
MeAsn-Phe + H	276.1333	276.1350	276.1320
CO-Lys-Phe + H	304.1630	304.1642	-
*Z*-MeAsn + H	-	-	318.1750
Phe-Lys-Ile + H	-	387.2375	387.2269
Lys-Ile-*Z* + H	-	-	429.2842
*Z*-Ile-Lys + H	401.2661	415.2674	429.2808
Ile-*Z*-MeAsn + H	403.2336	417.2517	431.2620
Phe-Lys-Ile-*Z* + H	-	562.3374	576.3330
Ile-*Z*-MeAsn-Phe + H	550.3302	564.3783	578.3556
Lys-CO-Lys-(Ile-*Z*) + H	575.3576	589.3531	603.3697
Lys-Ile-*Z*-MeAsn-Phe − NH_3_ + 2H	661.3682	675.3851	689.4008
Lys-Ile-*Z*-MeAsn-Phe + 2H	678.3934	692.4075	706.4232
CO-[Lys-Ile-*Z*-MeAsn-Phe] + H	704.3712	718.3935	732.4006
Lys-CO-Lys-(Phe)-(Ile-*Z*) + H	722.4142	736.4312	750.4436
Lys-CO-[Lys-Ile-*Z*-MeAsn-Phe] + H	850.4818	864.4972	878.5122

^a^ *Z* amino acid in the fourth position: Hph for compound **12**, MeHph for **14**, EtHph for **16**; ^b^ - not detected.

**Table 7:** Product ion spectra data for compounds **13**, **15**, and **17**.

Product Ion Assignment ^a^	13 (*m/z*)	15 (*m/z*)	17 (*m/z*)
Lys related fragment	70.0544	70.0619	70.0552
Lys fragment	84.0706	84.0731	84.0350
MeAsn immonium ion	101.0617	101.068	-
Phe immonium ion	120.0679	120.0748	-
Arg + H	175.1205	175.1184	175.1171
CO-Arg	201.0964	201.0966	201.0970
MeAsn-Phe − CO + H	-^b^	248.1435	248.1286
MeAsn-Phe + H	-	276.1298	276.1337
Phe-Lys-Ile + H	-	387.2358	387.2356
Ile-*Z*-MeAsn + H	403.7866	417.2410	-
Ile-*Z*-MeAsn-Phe + 2H	-	564.3384	-
Lys-Ile-*Z*-MeAsn-Phe − NH_3_ + 2H	-	-	689.3691
Lys-Ile-*Z*-MeAsn-Phe + 2H	678.4045	692.4084	706.4222
Arg-CO-Lys-(Phe)-(Ile-*Z*) + H	-	-	778.4472
Arg-CO-[Lys-Ile-*Z*-MeAsn-Phe] + H	878.4864	892.5023	906.5196

^a^ *Z* amino acid in the fourth position: Hph for compound **13**, MeHph for **15**, EtHph for **17**; ^b^ - not detected.

**Table 8:** Product ion spectra data for compounds **22** and **26**.

Product Ion Assignment ^a^	22 (*m/z*)	26 (*m/z*)
Lys fragment	84.0753	84.0768
Leu immonium ion	86.0924	86.0924
MeAsn immonium ion	101.0695	101.0677
Phe immonium ion	120.0810	120.0724
*Z* immonium ion	148.1124	162.1273
MeAsn-Phe − CO + H	248.1381	248.1369
MeAsn-Phe + H	276.1335	276.1326
CO-Lys-Phe + H	304.1667	- ^b^
Phe-Lys-Ile	387.2406	387.2370
Ile-*Z*-MeAsn + H	417.2486	431.2661
*Z*-Ile-Lys + H	415.2558	-
Ile-*Z*-MeAsn-Phe + 2H	564.3281	-
Leu-CO-Lys-Phe	433.2435	-
*CO-Lys-Phe-NMeAsn*	*431.1872*	-
Leu-CO-Lys-*N*MeAsn-Phe − CO + H	533.2997	533.2996
Leu-CO-Lys-*N*MeAsn-Phe	561.2995	561.2967
Leu-CO-Lys-(Ile)-(*N*MeAsn-Phe) − NH_3_+ H	-	657.3648
Leu-CO-Lys-(Ile)-(*N*MeAsn-Phe)	674.3823	674.3810
Leu-CO-Lys-*Z*-*N*MeAsn-Phe	736.3966	750.4096
[Lys-Ile-*Z*-MeAsn-Phe] + 2H	692.4077	706.4201
Leu-CO[Lys-Ile-*Z*-*N*MeAsn-Phe]	849.4872	863.5028

^a^ *Z* amino acid in the fourth position: MeHph for compounds **22** and EtHph for **26**; ^b^ - not detected.

**Table 9:** Product ion spectra data for compounds **25** and **27**.

Product Ion Assignment ^a^	25 (*m/z*)	27 (*m/z*)
Lys fragment	84.0714	84.0714
MeAsn immonium ion	101.0633	101.057
Phe immonium ion	120.0701	120.077
*Z* immonium ion	148.1074	162.1336
MeAsn-Phe − CO + H	248.1425	248.1362
MeAsn-Phe + H	276.1338	276.1333
CO-Lys-Phe + H	304.1570	- ^b^
Phe-CO-Lys + H	320.1601	320.1534
Phe-Lys-Ile + H	-	387.2388
Ile-*Z*-MeAsn + H	417.2582	-
*Z*-Ile-Lys + H	415.2709	-
Phe-CO-Lys-Phe + H	467.2262	-
Phe-CO-Lys-Phe-MeAsn + H	595.2844	595.2842
Phe-CO-Lys-Phe-MeAsn-*Z* + H	770.3866	-
Phe-CO-Lys-(Ile-*Z*)-(MeAsn) + H	736.3827	-
Phe-CO-Lys-Ile-*Z*-MeAsn + H	736.3827	-
Lys-Ile-*Z*-MeAsn-Phe	-	706.4299
Lys-Ile-*Z*-MeAsn-Phe + 2H	708.3705	708.3720
CO-[Lys-Ile-*Z*-MeAsn-Phe] + H	718.3895	732.4082
Phe-CO-(Lys-Ile-*Z*)-(Phe) + H	755.3925	767.3730
Phe-CO-[Lys-Ile-*Z*-MeAsn-Phe] + H	883.4695	897.4858

^a^ *Z* amino acid in the fourth position: MeHph for **25** and EtHph for **27**; ^b^ - not detected.

Thus, compound **12** (*m/z* 850.4818 [M + H]^+^) was characterized as Lys-CO[Lys-Ile-Hph-MeAsn-Phe]. As expected, the presence of Lys in the side chain generated a simple fragmentation pattern. Consequently, just two low intensity series of fragment ions that were assigned to two preferential primary cleavages were observed in the spectrum (Figure 4). However, enough information was generated to allow a tentative interpretation of the spectrum. The preferential fragmentation observed was attributed to the cleavage of the ureido linkage and the opening of the ring and indicated the presence of lysine in the side chain (*m/z* 678.3934). Further amino acid residue losses from this lineal ion (*i.e.*, acylium or similar) yielded a series of ions at *m/z* 550.3302, 403.2336, 304.1630, and 276.1333 (loss of Lys, Phe, Ile/Leu, and Hph, respectively). Another series of less abundant ions that were generated by the loss of *N*-methyl asparagine and phenylalanine was also observed at *m/z* 722.4142 (loss of MeAsn) and 575.3576 (loss of Phe). The dipeptide fragments at *m/z* 304.1630 and 276.1333 determined the partial sequence Hph-MeAsn-Phe and established the isoleucine/leucine at position 3 (*m/z* 403.2336) and the lysine that closed the ring (*m/z* 678.3934). Ions that are typically observed in the low *m/z* region were also present (Lys at *m/z* 84.0768, MeAsn at *m/z* 101.0702, Phe at *m/z* 120.0738).

Compounds **14** and **16**, which exhibited molecular mass increases of 14 and 28 Da, respectively, when compared to the compound at *m/z* 850.4818 [M + H]^+^, exhibited similar product ion spectra. Differences that were observed in the fragments containing the amino acid at position 4 suggested that this residue was either methylated (*m/z* 417.2517, 564.3783, 692.4075, and 736.4312) or ethylated (*m/z* 431.2620, 576.3330, 689.4008, and 750.4436). The existence of ions at *m/z* 148.1125 and 162.1213 that were attributed to the *N*-methylhomophenylalanine (*N*-MeHph) and *N*-ethylhomophenylalanine (*N*-EtHph) immonium ions, respectively, also supported these assignments.

Using similar reasoning, the remaining anabaenopeptins were subsequently characterized. Losses of 157 u that were observed for compounds **22** and **26**, losses of 191 u that were observed for compounds **25** and **27**, and losses of 200 u that were observed for compounds **13**, **15**, and **17** were used to identify the amino acid side chain as leucine, phenylalanine, or arginine, respectively. A fragmentation pattern similar to that described in the preceding paragraphs characterized the cyclic structures. As mentioned above, compounds with [M + H]*^+^* at *m/z* 878.5122, 892.5023, and 906.5196 exhibited low efficiency fragmentations, which are characteristic of oligopeptides containing strongly basic residues. When the collision energy used for fragmentation was increased to improve efficiency, the abundance of the fragment ions was compromised (Figure 4).

Four additional anabaenopeptins (**18**, **19**, **23**, **24**) were found in *Nostoc* sp. CENA352. Accurate mass measurement, isotopic profiles, and MS/MS spectra were conclusive to distinguish these compounds from previously described anabaenopeptins and characterize these compounds as new variants [36,53]. Structurally, all of these anabaenopeptins incorporated phenylalanine in the side chain position, as evidenced by the loss of a 191-u fragment from the protonated molecules (Table 10). Positions 5 and 6 of these four peptides were also conserved and were occupied by *N*-methyl-alanine and homotyrosine respectively. For the homovariant amino acids in position 4, either homophenylalanine (**18**, **19**) or homotyrosine (**23**, **24**) was proposed; for position 3, the commonly reported valine (**18** and **23**) or leucine/isoleucine (**19** and **24**) was proposed. For example, diagnostic low mass fragment ions that were observed in the product ion spectrum of the compound at *m/z* 842.4416 [M + H]^+^ (**23**) provided information about the amino acid residues, indicating the presence of lysine (*m/z* 84.0744), *N*-methyl-alanine (*m/z* 114.0515), phenylalanine (*m/z* 120.0736), homophenylalanine (*m/z* 134.0921), and homotyrosine (150.0887), while higher product ions suggested their sequence. Fragment ions at *m/z* 247.1440 and 263.1399 indicated the attachment of *N*-methyl-alanine to both homotyrosine and homophenylalanine, and subsequently established the sequence Val-Hph-MeAla for *m/z* 346.2124 and Lys-Val-Hph for *m/z* 387.2384. Taking into account the reported anabaenopeptins structures, the Phe-CO-[Lys-Val-Hph-MeAla-Hty] sequence was reasonably postulated. The intense fragment ion generated by the loss of a homophenylalanine residue at *m/z* 681.3591 and the less intense series of b-ions (*m/z* 596.3041, 497.2381, 320.1602, and 419.2283) were thus comprehensively attributed (Table 10).

**Table 10:** Product ion spectra data for compounds **18**, **19**, **23**, and **24**.

Product Ion Assignment ^a^	18 (*m/z*)	19 (*m/z*)	23 (*m/z*)	24 (*m/z*)
Lys fragment	84.0734	84.0757	84.0744	84.0749
MeAla + H − CO	114.0499	114.0557	114.0515	114.0514
Phe immonium ion	- ^b^	120.0736	120.0736	120.0742
Hph immonium	-	-	134.0921	134.0929
Htyr immonium	150.0907	150.0907	150.0887	150.0873
Hph-MeAla +H	-	-	247.1440	247.1485
HTyr-MeAla + H	263.1381	263.1394	263.1399	263.1391
*Y*-*Z*-MeAla + H	362.2138	376.2236	346.2124	360.2270
Lys-*Y*-*Z* + H	403.2366	417.2447	387.2384	401.2544
MeAla-Hty-Lys-*Y* + H	490.2942	-	490.3023	514.3045
Phe-CO-Lys-*Y* + H	-	-	419.2283	-
Phe-CO-Lys + 2H	-	-	320.1602	320.1605
Phe-CO-Lys-Hty + 2H	497.2336	497.2298	497.2381	497.2387
Phe-CO-Lys-*Y*-*Z* + H	-	610.3218	*596.3041*	610.3266
Phe-CO-Lys-Hty-MeAla + 2H	582.2993	582.2962	582.2918	582.2915
Lys-*Y*-*Z*-MeAla-Hty	665.3655	679.3793	649.3720	663.3819
Lys-*Y*-*Z*-MeAla-Hty + 2H	667.3786	681.3953	651.3873	665.4013
Phe-CO-Lys-(*Y*)-(Hty-MeAla) + 2H	681.3604	695.3756	681.3597	695.3756
CO-[Lys-*Y*-*Z*-MeAla-Hty] + H	693.3629	-	-	-
Phe-CO-[Lys-*Y*-*Z*-MeAla-Hty] + H − CO	830.4384	844.4575	814.4502	828.4634
Phe-CO-[Lys-*Y*-*Z*-MeAla-Hty] + H − H_2_O	840.4385	854.4465	824.4377	838.4398
Phe-CO-[Lys-*Y*-*Z*-MeAla-Hty] + H	858.4405	872.4544	842.4416	856.4561

^a^ *Y* amino acid in the third position: Val for compounds **18** and **23** and Ile for **19** and **24**; *Z* amino acid in the fourth position: Hty for **18** and **19** and Hph for **23** and **24**; ^b^ - not detected.

In addition, a pair of unusual tryptophan-containing anabaenopeptins (**20**–**21**) was also detected at *m/z* 803.4417 [M + H]^+^ and 803.4425 [M + H]^+^, exclusively in the genus *Brasilonema* sp. (CENA360 and CENA382). As this pair of compounds exhibited similar protonated molecules and product ion spectra but differed in retention times, these compounds were classified as diastereoisomers. The structures of these compounds were postulated in accordance with the fragment ions listed in Table 11. According to our literature search, prior to our work, the occurrence of tryptophan-containing anabaenopeptins in cyanobacteria was limited to the genus *Tychonema* sp. [64]. The structurally related compounds isolated from this genus, the brunsvicamides A–C, inhibit tyrosine phosphatase B of *Mycobacterium tuberculosis* (MptpB) [64] and are highly selectivity inhibitors for human leukocyte elastase (HLE) [65]. However, those peptides and the compounds reported here differ in their amino acid sequences. Unlike the brunsvicamides, the postulated anabaenopeptins contain tryptophan in position 4 and *N*-methyl-alanine in the methylated amino acid position. The structures of the compounds described in this study are more closely related to a synthetic brunsvicamide analog described by Walther *et al.* [66], which was found to be an inhibitor of carboxypeptidase A. Thus, further biological tests of these compounds are warranted.

**Table 11:** Product ion spectra data for compounds **20** and **21**.

Product Ion Assignment	20 (*m/z*)	21 (*m/z*)
Lys fragment	84.0730	84.0721
MeAla + H − CO	114.0519	114.0504
Phe immonium ion	120.0833	120.0747
Trp fragment	130.0645	130.0607
Lys-Ile + H	240.1738	240.1752
Trp-MeAla + H	272.1384	272.1429
Ile-Trp-MeAla + H	385.2229	385.2238
CO-Lys-Phe + H	320.1599	320.1602
Val-CO-Lys-Ile + H	385.2229	385.2211
CO-Lys-Phe-MeAla + H	405.2128	405.2084
Val-CO-Lys-Ile + H	419.2214	419.2240
Val-CO-Lys-Phe-MeAla + H	504.2829	504.2806
Val-CO-Lys-Phe-Ile + H	532.3069	532.3204
Val-CO-Lys-(Ile)-(Phe-MeAla) + H	617.3706	617.3686
Lys-Ile-Trp-MeAla-Phe + H	658.3680	658.3493
Lys-Ile-Trp-MeAla-Phe + 2H	660.3879	660.3833
CO-Lys-Ile-Trp-MeAla-Phe + H	686.3690	686.3681
Val-CO-Lys-(Ile-Trp)-(Phe) + H–CO_2_	674.3749	674.3970
Val-CO-[Lys-Ile-Trp-MeAla-Phe] + H − CO_2_	759.4538	759.4521
Val-CO-[Lys-Ile-Trp-MeAla-Phe] + H − CO	775.4563	775.4595
Val-CO-[Lys-Ile-Trp-MeAla-Phe] + H − H_2_O	785.4408	785.4278
Val-CO-[Lys-Ile-Trp-MeAla-Phe] + H	803.4417	803.4425

### 2.3. Cyanopeptolins

Eleven new cyanopeptolins with protonated molecules at *m/z* 984.5381 (**29**), 984.5398 (**33**), 998.5531 (**31**), 998.5549 (**35**), 998.5564 (**36**), 1002.5031 (**28**), 1004.4831 (**30**), 1012.5737 (**37**), 1018.5011 (**32**), 1026.5858 (**38**), and 1032.5170 (**34**) were present in the extracts from *Desmonostoc* strains CENA371 and CENA386. Although no single diagnostic fragment ion can be used to identify this family of peptides, series of fragments related to the conserved position 4 (3-amino-6-hydroxy-2-piperidone amino acid (Ahp) could be used to identify these compounds [23]. Under our experimental conditions, doubly charged ions ([M − H_2_O + 2H]^2+^) with an abundance comparable to that of protonated molecules were also observed in the mass spectra of all detected cyanopeptolins. MS and MS/MS analyses suggested closely related structures for all of these cyanopeptides (Table 12, Table 13 and Table 14). As the most common structural feature, these cyanopeptolins contained *N*-acetyl-proline-glutamine as side chain, with the fifth position occupied by either a dimethylated tyrosine or chlorinated-methylated tyrosine. Valine and leucine alternated in positions 4 and 6.

**Table 12:** Product ion spectra data for compounds **30**, **32**, **34**, and **37**.

Product Ion Assignment ^a^	30 (*m/z*)	32 (*m/z*)	34 (*m/z*)	37 (*m/z*)
Pro immonium	70.0542	70.0595	70.0571	70.0541
Val immonium	-^b^	72.0692	-	-
Leu immonium	-	-	86.0900	86.0935
*N*AcPro immonium	112.0695	112.0732	112.0679	112.0679
*N*AcPro + H	140.0623	140.0773	140.0678	140.0706
*N*Me-*R*^1^-Tyr immonium	184.0572	184.0486	184.0523	164.1052
Leu-Ahp − H_2_O − CO + H	-	181.1263	181.1290	181.1300
Leu-Ahp − H_2_O + H	209.1239	-	209.1257	209.1286
Ac-Pro-Gln + H	268.1207	268.1276	268.1270	268.1271
Ac-Pro-Gln-Thr − H_2_O + H	351.1656	351.1645	351.1638	351.1633
*N*Me-*R*^1^-Tyr-*X*-Ahp − H_2_O + H	406.1509	406.1515	420.1648	400.2181
AcPro-Gln-Thr-Leu − H_2_O + H	464.2465	464.2447	464.2467	464.2468
AcPro-Gln-Thr-Leu-Ahp-*X* − 2H_2_O + H	-	-	-	672.3922
AcPro-Gln-Thr-Leu-Ahp-*X* − H_2_O + H	-	-	-	690.3777
Thr-Leu-Ahp-*X*-*N*Me-*R*^1^-Tyr-*Z* − H_2_O + 2H	727.0780	733.3705	747.3811	727.4318
AcPro-Gln-Thr-(Z-*N*Me-*R*^1^-Tyr)-(Leu) + H	-	-	806.3871	786.4305
AcPro-Gln-Thr-Leu-Ahp-*Z*-*N*Me-*R*^1^Tyr − H_2_O + H	-	-	901.4207	881.4576
AcPro-Gln-[Thr-Leu-Ahp-*X*-*N*Me-*R*^1^-Tyr-*Z*] − H_2_O +H	986.4693	1000.4815	1014.5018	994.5553
AcPro-Gln-[Thr-Leu-Ahp-*X*-*N*Me-*R*^1^-Tyr-*Z*] + H	1004.4831	1018.5011	1032.5170	1012.5737

^a^ *X* amino acid in the fourth position: Val for compounds **30** and **32** and Leu for **34**, **36-37**; *Z* amino acid in the sixth position: Val for **30** and **36** and Leu for **32**, **34**, and **37**; *R*^1^: *O*-methyl for compounds **36** and **37**; Cl for compounds **30**, **32**, and **34**; ^b^ - not detected.

**Table 13:** Product ion spectra data for compounds **29**, **31**, **33**, and **35**–**36**.

Product Ion Assignment ^a^	29 (*m/z*)	31 (*m/z*)	33 (*m/z*)	35 (*m/z*)	36 (*m/z*)
Pro immonium	-^b^	-	-	-	70.0563
Val immonium	-	-	-	-	-
Leu immonium	86.0924	-	-	-	86.0876
*N*AcPro immonium	112.0755	112.0755	112.0755	112.0688	112.0679
*N*AcPro + H	140.0618	140.0693	140.0693	140.0693	140.0696
*N*Me-*R*^1^-Tyr immonium	164.1024	150.0907	164.1024	164.1024	164.1052
Leu-Ahp − H_2_O − CO + H	181.1353	181.1267	-	-	181.1283
Leu-Ahp − H_2_O + H	209.1243	209.1335	-	209.1244	209.1331
Ac-Pro-Gln + H	268.1258	268.1258	-	268.1258	268.1207
Ac-Pro-Gln-Thr − H_2_O + H	351.1645	351.1637	351.1638	351.1658	351.1642
*N*Me-*R*^1^-Tyr-*X*-Ahp − H_2_O + H	386.2025	386.2008	386.2064	386.2034	400.2250
AcPro-Gln-Thr-Leu − H_2_O + H	464.2439	464.2464	464.2493	464.2495	464.2549
AcPro-Gln-Thr-Leu-Ahp-*X* − 2H_2_O + H	-	-	-	658.3446	-
AcPro-Gln-Thr-Leu-Ahp-*X* − H_2_O + H	-	-	-	-	-
Thr-Leu-Ahp-*X*-*N*Me-*R*^1^-Tyr-*Z* − H_2_O + 2H	699.4064	713.4158	699.4065	713.4195	713.3971
AcPro-Gln-Thr-(Z-*N*Me-*R*^1^-Tyr)-(Leu) + H	-	772.3942	-	786.4170	-
AcPro-Gln-Thr-Leu-Ahp-*X*-*N*Me-*R*^1^Tyr − H_2_O + H	-	-	-	867.4460	867.4647
AcPro-Gln-[Thr-Leu-Ahp-*X*-*N*Me-*R*^1^-Tyr-*Z*] − H_2_O +H	966.5061	980.5489	966.5144	980.5472	980.5382
Ac-Pro-Gln-[Thr-Leu-Ahp-*X*-*N*Me-*R*^1^-Tyr-*Z*] + H	984.5381	998.5531	984.5398	998.5550	998.5564

^a^ *X* amino acid in the fourth position: Val for compounds **29**, **33** and **35** and Leu for **31** and **36**; *Z* amino acid in the sixth position: Val for **29**, **33** and **36** and Leu for **31**, **35** and **37**; *R*^1^: *O*-methyl for compounds **29**, **31**, **33**, **35** and **36**; ^b^ - not detected.

**Table 14:** Product ion spectra data for compound **28** and **38**.

Product Ion Assignment ^a^	28 (*m/z*)	38 (*m/z*)
Pro immonium	- ^b^	-
Leu immonium	86.08763	-
*Y* immonium	-	126.0845
*Y* + H	-	154.0743
*N*Me-*R*^1^-Tyr immonium	184.0486	164.1060
Leu-Ahp − H_2_O − CO + H	-	-
Leu-Ahp − H_2_O + H	209.1239	-
*Y*-Gln + H	268.1207	288.1510
Ac-Pro-Gln-Thr − H_2_O + H	321.1521	365.1662
*N*Me-*R*^1^-Tyr-*X*-Ahp − H_2_O + H	420.1585	400.2211
*Y*-Gln-Thr-Leu − H_2_O + H	434.2416	478.2652
*Y*-Gln-Thr-Leu-Ahp-*X* − 2H_2_O + H	-	686.5626
*Y*-Gln-Thr-Leu-Ahp-*X* − H_2_O + H	-	-
Thr-Leu-Ahp-*X*-*N*Me-*R*^1^-Tyr-*Z* − H_2_O + 2H	-	727.4402
*Y*-Gln-Thr-(Z-*N*Me-*R*^1^-Tyr)-(Leu) + H	-	800.4147
*Y*-Gln-Thr-Leu-Ahp-*X*-*N*Me-*R*^1^Tyr − H_2_O + H	-	-
*Y*-Gln-[Thr-Leu-Ahp-*X*-*N*Me-*R*^1^-Tyr-*Z*] − H_2_O +H	984.4914	1008.5481
*Y*-Gln-[Thr-Leu-Ahp-*X*-*N*Me-*R*^1^-Tyr-*Z*] + H	1002.5031	1026.5858

^a^ *Y* exocyclic amino acid in position 1: methyl-dehydroproline (Mdhp) for compound **28** and *N*-propanoyl-proline for compound **38**; *X* amino acid in the fourth position: Leu; *Z* amino acid in the sixth position Leu; *R*^1^: Cl for compound **28** and *O*-methyl for compound **38**; ^b^ - not detected.

This family of cyclic peptides with high structural variability featured a ring formed by six amino acids and a side chain of different lengths and composition [3]. An ester bond between the hydroxyl group of the threonine in position one and the carboxyl group of the terminal amino acid cyclizes the ring. The threonine amino acid in position 1 is occasionally replaced by 3-hydroxy-4-methylproline [67,68]. The 3-amino-6-hydroxy-2-piperidone amino acid (Ahp) always occupied position 3, while a methylated aromatic amino acid and other neutral amino acids are found in positions 5 and 6, respectively. The highly variable side chain may contain an aliphatic fatty acid or a glyceric acid, which is attached either directly to the threonine in position 1 or through one or two amino acids [3]. This family of peptides is often described as protease inhibitors [69,70,71,72,73]. Cyanopeptolins have been isolated mostly from *Microcystis* [71,74,75] but also from other genera such as *Lyngbya* [76,77], *Nostoc* [67,78], *Oscillatoria* [79,80], *Planktothrix* [60,81], *Scytonema* [82], or *Symploca* [72].

**Figure 5:**
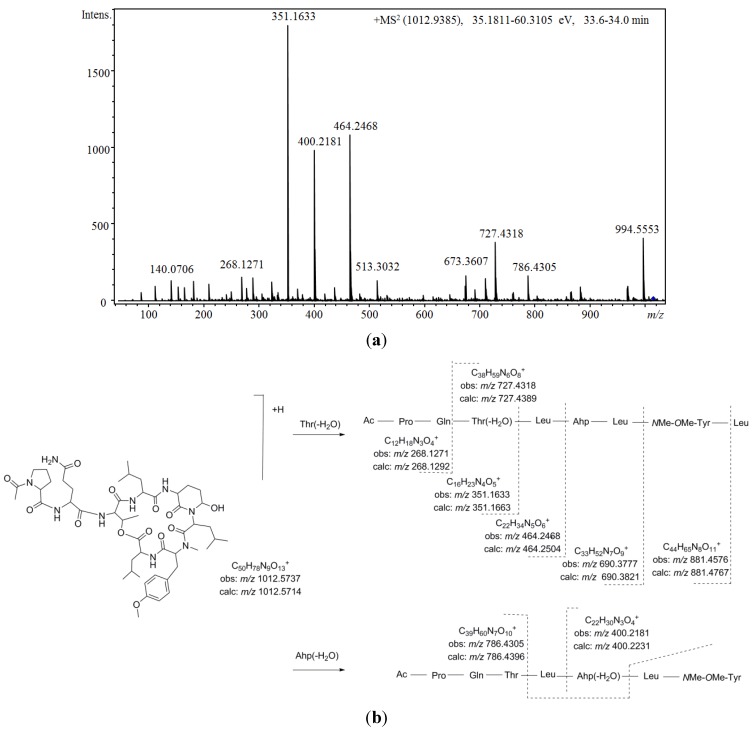
(**a**) Product ion spectrum for [M + H]^+^ of compound **37** and (**b**) its predicted fragmentation pattern. Conditions as described in the experimental section.

Figure 5 shows the proposed depsipeptide cyclic structure for compound **37** (Ac-Pro-Gln[Thr-Leu-Ahp-Leu-*N*Me-OMe-Tyr-Leu]) and its predicted fragmentation pattern. The most abundant ions observed in the product ion spectrum of this compound were attributed to the loss of amino acid residues from the *C*-terminus (*m/z* 881.4576, 690.3777, 464.2468, 351.1633, and 268.1271) of the dehydrated protonated molecule at *m/z* 994.5553. This precursor ion was suggested to be generated by the cleavage of the ester linkage accompanied by the dehydration of the Thr [34]. Further loss of the acetyl-proline-glutamine side chain from this linear ion was also noted (*m/z* 727.4318). Simultaneously, evidence of dehydration at the Ahp position was observed at *m/z* 400.2184 and 786.4305. An inspection of the low *m/z* region of the spectrum also supported the proposed structure, revealing the presence of ions associated with the amino acids Ahp (*m/z* 209.1286 and 181.1300), *N*Me*O*MeTyr (*m/z* 164.1052), AcPro (*m/z* 112.0679 and 140.0703), Leu (*m/z* 86.0979), and Pro (*m/z* 70.0563). To our knowledge, the *N*-acetyl-proline-glutamine side chain has not been 

Comparing the aforementioned data with those obtained for compound **36**, a highly similar structure was deduced. Differences of 14 u in the protonated molecules and a fragment ion at *m/z* 713.3971 in combination with the existence of product ions at *m/z* 464.2549 and 400.2250 indicated that the leucine attributed to position 6 in compound **37** was replaced by valine. In addition, two other isobaric compounds were observed at earlier retention times (**31** at 30.0 min, and **35** at 31.1 min). Structural differences from compound **36** were proposed based on mass data (Table 13). Thus, monomethylated tyrosine was suggested for compound **31** (*m/z* 772.3942, 713.3942, and 150.0907) and a valine residue occupying the fourth position instead of the sixth position was suggested for **35** (*m/z* 867.4460, 786.4170, 713.4195, and 164.1024). Furthermore, two other minor compounds (**29** and **33**) with molecular masses 14 Da lower than the compounds mentioned above and with similar product ion spectra were also detected. Based on the mass results (*m/z* 966.5061/966.5144 and 699.4064/699.4065), the incorporation of valine at positions 4 and 6 was postulated for this pair of diastereoisomers. Simultaneously with these compounds, another congener was observed at *m/z* 1026.5858 [M + H]^+^ (**38**). Propanoylation of the proline residue instead of the more commonly detected acetylation was suggested for this compound on the basis of its product ion spectrum, which differed in product ions containing the side chain (*m/z* 365.1662, 478.2652, and 686.5626) in combination with the existence of product ions at *m/z* 126.0845 and 154.0743 (attributed to *N*-propanoyl-proline).

Similarly, four additional compounds (**28**, **30**, **32**, and **34**) exhibited fragmentation patterns highly similar to that of compound **37**. While most of the mass spectrum remained conserved, fragment ions containing the original dimethylated tyrosine amino acid exhibited *m*/*z* shifts when compared to that of the model compound. Additionally, the isotopic pattern of these fragment ions in conjunction with that of the protonated molecule revealed the presence of a chlorine atom in their structures. For compound **34**, these fragment ions shifted 20 u (*m/z* 901.4207, 806.3871, 747.3811, 420.1648, and 184.0523), leading us to propose the chlorination of a methylated tyrosine at position 5. For compound **32**, shifts of 20 and 14 u were observed (*m/z* 733.3705, 406.2447, and 184.0486), suggesting that in addition to the modification mentioned above, a substitution of leucine with valine at position 4 also occurred. Based on the same logic, a structure similar to compound **34**, just with the leucine residue at position 6 replaced by valine, was proposed for compound **30**. Finally, for compound **28**, differences of 30 u in product ions containing the side chain were observed in comparison to compound **34** (*m/z* 321.1521 and *m/z* 434.2416). Thus, the same cyclic peptide was proposed for compound **28** with the side chain tentatively attributed to methylated-dehydroproline-glutamine (Mdhp-Gln) or other analogs. The chlorinated and methylated tyrosine amino acid proposed for position 5 was quite unusual and has only been observed in a small number of cyanopeptolins [71,80].

The structural similarity of these compounds to other cyanopeptolins with observed protease inhibitory activity warrants further bioactivity assays. Trypsin inhibitory selectivity was suggested to be related to the existence of basic residues adjacent to Ahp, while chymotrypsin selectivity was proposed to be related to hydrophobic residues. Additionally, residues in other positions as side chains or in the fifth position appear to influence this activity [5,72,83]. These assays will be able to establish the influence of the particular properties of these compounds on the selectivity and potency of these and other activities.

## 3. Experimental Section

### 3.1. Strains of Cyanobacteria and Cultivation Conditions

The 40 surveyed cyanobacterial strains belong to the culture collection of the Center for Nuclear Energy in Agriculture Collection/University of São Paulo (CENA/USP), Brazil. All strains were isolated from the leaves of four plant species—*Euterpe edulis*, *Guapira opposita*, *Garcinia gardneriana*, and *Merostachys neesii*—that were collected in two regions of the Parque Estadual Serra do Mar (Southeastern Brazil). The isolates (three Choococcales, 13 Pseudanabaenales, and 24 Nostocales) were previously identified using both morphological analysis and phylogenies based on the 16S rRNA gene [30] (Table 1). The cultures of cyanobacteria were maintained in liquid BG11 medium under white fluorescent light (30 mmol photons·m^−2^·s^−1^) with a 14:10 h light/dark cycle at 25 ± 1 °C under constant agitation (150 rpm) for 21 days. The cells were then concentrated by centrifugation (7000× *g*, 5 min), washed three times in saline solution (NaCl 0.8%), and re-inoculated into 500-mL flasks containing 200 mL of medium and cultured for a further 21 days.

### 3.2. Extraction of Peptides

The cell suspensions were concentrated by centrifugation at 7000× *g* for 5 min and lyophilized (SNL216V, Thermo Electron Corporation). The lyophilized cells (10 mg) were extracted three times with 70% methanol via probe sonication (amplitude of 30%, 2 min, Soni Omni Disruptor), centrifuged (9000× *g*, 4 °C, 10 min) and concentrated under a stream of nitrogen (TE-concentrator, Techal). The residue was redissolved in 1 mL of methanol:water (50/50, v/v) for LC/MS analysis.

### 3.3. LC/MS Analyses

Analyses were carried out on a Shimadzu Prominence Liquid Chromatography system coupled to a quadrupole time-of-flight mass spectrometer (Micro TOF-QII; Bruker Daltonics, MA, USA) with an ESI interphase. Separations were achieved using a Luna C18 (2) column (250 mm × inner diameter 3:00, 5 μm) (Phenomenex, Torrance, CA, USA) protected with a guard column of the same material. Samples (5 μL) were eluted using a mobile phase A (water, 0.1% formic acid, and 5 mM ammonia formate) and a mobile phase B (acetonitrile). The gradient increased linearly from 5% to 90% B over 50 min at a flow rate of 0.2 mL/min. The ionization source conditions were as follows: positive ionization, capillary potential of 3500 V, temperature and flow of drying gas (nitrogen) of 5 mL/min and 300 °C, respectively, nebulizer pressure of 35 psi. Mass spectra were acquired using electrospray ionization in the positive mode over the range of *m/z* from 50 to 3000. The Q/TOF instrument was operated in scan and AutoMS/MS mode, performing MS/MS experiments on the three most intense ions from each MS survey scan. Three collision-induced dissociation CID experiments were performed by varying the collision energies from 30 to 70 to produce many fragment ions of high abundance. The collision energies for fragmentation were as follows. Experiment 1: *m/z* 0–500, (a) 35 eV (single charged precursor ions) and (b) 25 eV (doubly charged charge); *m/z* 500–1000, (a) 50 eV and (b) 40 eV; and *m/z* 1000–2000, (a) 70 eV and (b) 50 eV. Experiment 2: *m/z* 0–500 (a) 50 eV and (b) 40 eV; *m/z* 500–1000, (a) 65 eV and (b) 55 eV; and *m/z* 1000–2000; (a) 85 eV and (b) 65 eV. Experiment 3: *m/z* 0–500, (a) 35 eV and (b) 25 eV; *m/z* 500–1000, (a) 65 eV and (b) 55 eV; and *m/z* 1000–2000, (a) 70 eV and (b) 50 eV with a 75–150% collision energy sweep. The mass spectrometer was calibrated externally with a 10 mM sodium formate cluster solution consisting of 10 mM sodium hydroxide and 0.1% formic acid in water-isopropanol 1:1 (v:v*).* The accurate mass data were processed using Data Analysis 4.0 software (Bruker Daltonics, Bremen, Germany) which provided a ranking of possible elemental formulae (EF) by using the SmartFormulaEditorTM. For each EF, error (deviations between the measured and theoretical mass of a given sum formula) and sigma value (comparisons of the theoretical and the measured isotope pattern of a given formula) are calculated [65]. The confirmation of the elemental formula was based on the widely accepted thresholds of 5 ppm and 20 m Sigma. Every experiment was run in triplicate.

### 3.4. Chemical

HPLC grade methanol and acetonitrile from J.T. Baker (USA). Ammonium formate and formic acid, both for mass analyses, were obtained from Fluka (Germany). 

## 4. Conclusions

In the present study, liquid chromatography coupled to a quadrupole time-of-flight mass spectrometer and equipped with an ESI interface was successfully applied to study in depth the cyanopeptide composition of 40 cyanobacterial strains from the Brazilian Atlantic Forest. This approach allowed us to tentatively identify 38 peptides, of which 37 had not been previously described in literature, including aeruginosins, anabaenopeptins, and cyanopeptolins. Based on the mass accuracy data in scan and product ion spectra in combination with the isotopic pattern of the deprotonated and product ions, a planar structure was postulated for each of the detected peptides. In addition to the recently reported aeruginosin 865, 10 novel structural variants were described here. Either hexose or glucuronic acid and butanoic, hexanoic, heptanoic, or octanoic acid *O*-linked to a Choi motif were observed. With respect to anabaenopeptins, this study led to the characterization of 16 anabaenopeptins. Among those anabaenopeptins, two tryptophan-containing anabaenopeptins and 10 additional anabaenopeptins that incorporated the amino acid *N*-methyl asparagine were identified. Furthermore, on several occasions, ethylation was postulated for the homovariant amino acid in position four. With respect to cyanopeptolins, 11 new variants were characterized. An *N*-acetyl proline-glutamine side chain mostly featured these compounds. Additionally, four of these compounds contained the unusual chlorinated *N-*methylated tyrosine. These results highlight the potential of LC-ESI-QTOF-MS for peptide characterization purposes in complex mixtures from small quantities of material. However, the combination of MS to other techniques such as X-ray crystallography or nuclear magnetic resonance (NMR) applied to the pure compounds is necessary for a complete spectroscopic characterization of the proposed peptide structures including the stereochemistry determination.

Among the surveyed strains of cyanobacteria, only nine strains were observed to produce cyanopeptides (three Nostoc sp., two Desmonostoc sp., and four *Brasilonema* sp.). However, a highly diverse array of new peptide variants was revealed in the producer strains, which emphasizes the potential of underexplored environments as a source of bioactive compounds.
